# DTCR: generating realistic, diverse, and epitope-specific T cell receptor sequences via a discrete diffusion model

**DOI:** 10.1093/bib/bbag438

**Published:** 2026-08-27

**Authors:** Haoyan Wang, Tianyi Zang, Yadong Liu

**Affiliations:** Faculty of Computing, Harbin Institute of Technology, No. 92 West Dazhi Street, Nangang District, Harbin, Heilongjiang 150001, China; Faculty of Computing, Harbin Institute of Technology, No. 92 West Dazhi Street, Nangang District, Harbin, Heilongjiang 150001, China; Faculty of Computing, Harbin Institute of Technology, No. 92 West Dazhi Street, Nangang District, Harbin, Heilongjiang 150001, China; Zhengzhou Research Institute, Harbin Institute of Technology, No. 26 Longyuan East Seventh Street, Zhengdong New District, Zhengzhou, Henan 450000, China

**Keywords:** T cell receptors (TCRs), TCR sequence generation, discrete diffusion model, immunotherapy

## Abstract

T lymphocytes are central to adaptive immunity, utilizing clonally distributed T cell receptors (TCRs) to recognize peptide-major histocompatibility complex ligands with high specificity. The immense diversity and precise antigen recognition of TCRs are critical for mounting effective immune responses. However, conventional experimental approaches for TCR discovery and optimization remain constrained by a narrow range of targetable epitopes, tumor immune evasion, and prohibitive costs. These bottlenecks hinder the broad clinical translation of TCR-T immunotherapies and create an urgent demand for advanced computational frameworks for the *de novo*, controllable generation of functional, epitope-specific TCR sequences. Here, we introduce DTCR, the first discrete diffusion-based generative model for epitope-specific TCR sequence generation. Unlike existing models, DTCR emulates the natural TCR amino acid substitution dynamics through a discrete corruption scheme and incorporates a binding specificity prediction module to guide controllable generation. This integrated framework not only enhances binding specificity and sequence diversity, but also enables flexible TCR generation tailored to target epitopes. DTCR outperforms state-of-the-art epitope-specific TCR generation models (GRATCR, an epitope‑specific TCR generation model, and TCR-TRANSLATE) in binding specificity, achieving relative improvements of 7.56%, 17.21%, 7.60%, 0.45%, and 6.86% over the second-best model TCR-TRANSLATE, as evaluated by five widely used TCR specificity prediction tools (Physics-Inspired Sliding Transformer (PISTE); TCR–Epitope Interaction Modelling (TEIM); ERGO, a peptide‑TCR matching prediction tool; epiTCR, a Random Forest‑based TCR‑peptide binding predictor; and NetTCR-2.0, a convolutional neural network‑based TCR‑peptide binding prediction tool), respectively. Furthermore, DTCR-generated TCRs exhibit stronger sequence diversity, better consistency with natural biological conservation patterns, and greater binding interface burials that support enhanced binding interface stability. The application of DTCR in the development of novel immunotherapies holds significant promise, offering a powerful tool for accelerating the discovery of effective and specific TCRs and advancing personalized medicine. The source code is available at: https://github.com/skybluewhy/DTCR.

## Introduction

T cells are essential components of the adaptive immune system, primarily responsible for recognizing and responding to specific antigens presented by peptide-major histocompatibility complexes (pMHCs) via the T cell receptor (TCR) [1]. The TCR is a transmembrane heterodimeric protein complex expressed on the T cell surface, and it mediates specific binding to pMHC molecules [2]. For the predominant αβ TCR heterodimer, each chain contains three complementarity-determining regions (CDRs): CDR1, CDR2, and CDR3. Among them, CDR1 and CDR2 primarily interact with the MHC molecule, while the hypervariable CDR3 region (especially the TCR β chain CDR3) is the core determinant of antigenic peptide recognition and binding specificity [3]. The remarkable diversity of TCRs arises from somatic variable, diversity, and joining (V(D)J) gene recombination, where the random assembly of gene segments can theoretically generate ~${10}^{15}$–${10}^{20}$ distinct receptor sequences [4]. This enormous sequence diversity is the foundation for the broad antigen recognition capacity of the adaptive immune system, but also poses a formidable challenge to the rational discovery and optimization of epitope-specific TCRs.

Cancer immunotherapy has evolved to encompass multiple innovative strategies, including monoclonal antibodies, immune checkpoint inhibitors, and adoptive cell therapy (ACT). These strategies are designed to activate and enhance the immune system’s ability to recognize and eliminate tumors [5]. Despite their promising advancements, current immunotherapies face significant challenges, including inconsistent efficacy, a narrow range of targetable antigens, complex tumor evasion mechanisms, and severe treatment-related adverse events, all of which substantially constrain their widespread clinical translation [6]. In particular, TCR-T cell therapy, a core branch of ACT, faces a critical bottleneck in the design of TCRs with optimal specificity and therapeutic efficacy.

To complement and accelerate traditional TCR discovery and optimization, there is an urgent need to develop advanced computational methodologies for predicting TCR–pMHC interactions, as well as for *de novo* generation and targeted optimization of functional TCR sequences. Diverse generative frameworks have been established for global TCR repertoire sequence modeling, including selection-based probabilistic models [7], variational autoencoders [8], and generative adversarial networks [9]. These methods excel at capturing the overall sequence distribution and statistical patterns of natural human TCR repertoires, yet they are not specifically tailored for the conditional generation of epitope-specific TCRs. With the advent of Transformer-based large language models (e.g. Generative Pre‑trained Transformer (GPT)), the field has witnessed transformative advancements in protein sequence representation learning and generation. Building on this foundation, researchers have developed specialized TCR language models that capture the intrinsic features and functional patterns of TCR sequences, exemplified by Bidirectional Encoder Representations from Transformers (BERT)‑based TCR-BERT [10] and T-cell receptor and Peptide Interaction Recognizer (TAPIR) [11]. Leveraging these pretrained TCR language models as backbones, subsequent studies have further extended this paradigm to epitope-specific TCR generation, with representative methods including Epitope‑Receptor‑Transformer (ERTransformer) [12] and GRATCR [13], an epitope‑specific TCR generation model. More recently, translation-based models have also been introduced for epitope-specific TCR generation, treating it as a sequence-to-sequence translation task [14].

Existing epitope-specific TCR generation methods predominantly rely on natural language modeling paradigms. These approaches tend to oversimplify TCR sequences as sentences in natural language, and while pretrained TCR language models can capture basic sequence statistical patterns, the unique evolutionary and biological constraints of TCRs (such as natural amino acid substitution dynamics and antigen-driven specificity selection constraints) are not actively integrated into the conditional generation framework. This oversight may lead to generated sequences that fail to recapitulate the core biological features of naturally occurring functional TCRs, thereby reducing their biological plausibility. Moreover, state-of-the-art TCR–epitope prediction models, validated by extensive experiments, can effectively extract key features and underlying patterns of TCR–epitope interactions. How to utilize the mechanistic insights learned by these prediction models to enhance the performance of TCR generation models remains a challenge.

**Figure 1:**
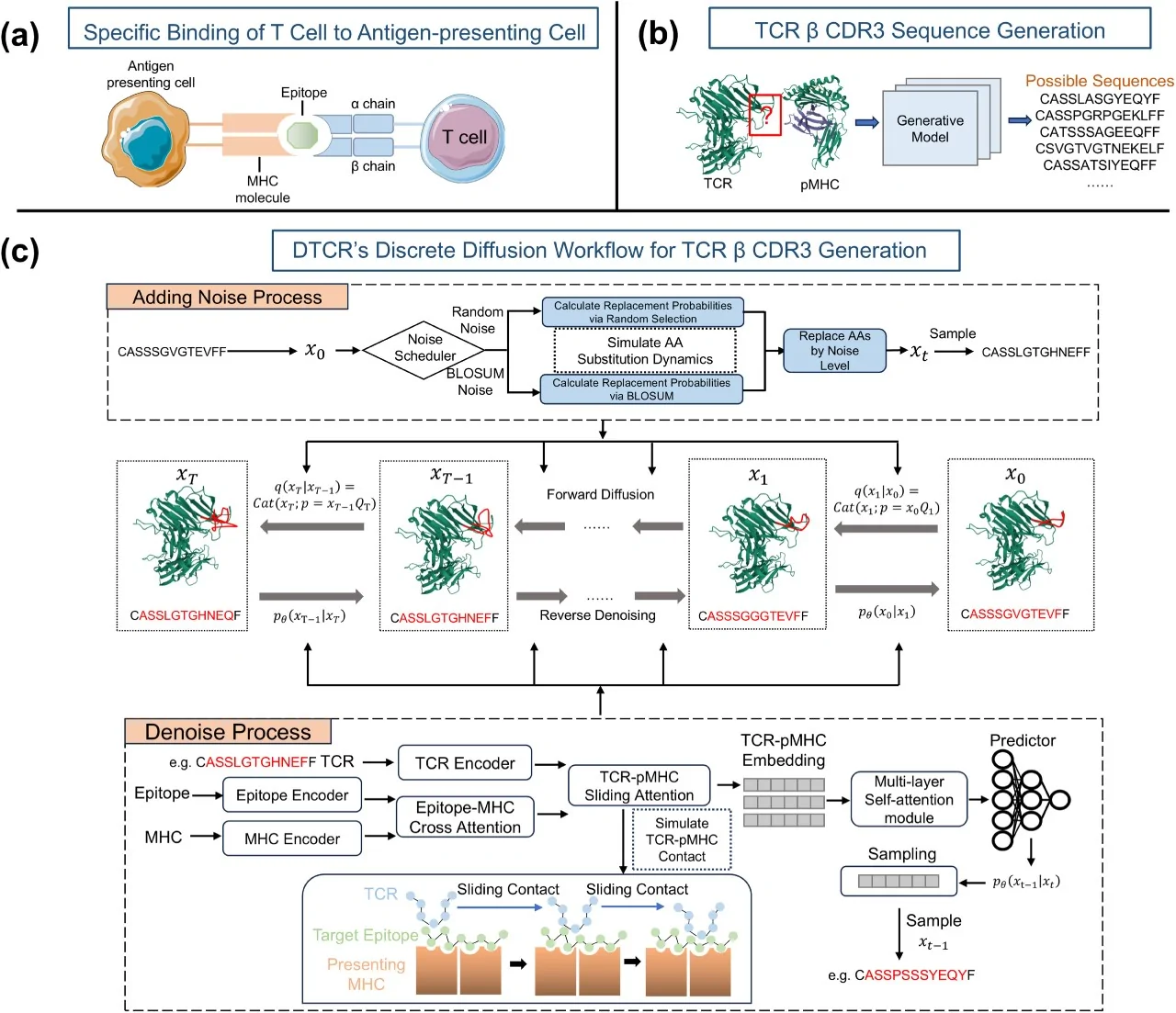
Overview of DTCR; (a) specific binding of T cell to antigen-presenting cell (APC); the TCR on the T cell surface specifically recognizes and binds to the pMHC presented on the APC; this interaction is crucial for initiating T cell activation and the subsequent immune response; (b) TCR β CDR3 sequence generation; the generation focuses on the hypervariable CDR3 region of the TCR beta chain, which is critical for antigen recognition; traditional methods face challenges in generating diverse and specific sequences due to the vast combinatorial space of TCRs (~${10}^{15}$–${10}^{20}$ possible sequences); DTCR addresses this by leveraging evolutionary and functional constraints to guide sequence generation; (c) DTCR’s discrete diffusion workflow for TCR β CDR3 generation; the model defines a discrete Markov forward process for sequence corruption through two distinct transition matrices: one based on random transitions and the other employing the BLOSUM matrix; these transition matrices control the process of amino acid identity changes, simulating natural amino acid substitution dynamics; by integrating an advanced TCR-epitope binding prediction model, DTCR constructs a denoising neural network to predict amino acid state changes at each time step; ultimately, DTCR can initiate from random amino acid sequences and generate new epitope-specific TCR sequences through a reverse denoising process conditioned on target pMHC.

Diffusion models [15] have been widely adopted for generative modeling due to their powerful fitting capacity and flexible probabilistic framework. However, traditional Gaussian diffusion models face significant limitations when applied to discrete sequence data, particularly in protein sequence modeling. In contrast, discrete diffusion models operate directly in discrete state spaces, which enable them to more effectively capture and maintain the inherent discrete properties of biological sequences [16, 17]. Building on these advances, we propose a TCR generation model based on discrete diffusion (DTCR). Figure 1 provides a comprehensive overview of DTCR, including T cell–pMHC recognition (Fig. 1a), TCR β CDR3 generation (Fig. 1b), and the discrete diffusion generation workflow (Fig. 1c). Specifically, the model defines a discrete Markov forward process for sequence corruption through two distinct transition matrix strategies: one based on random transitions, and the other utilizing the Blocks Substitution Matrix (BLOSUM) derived from amino acid substitution frequencies. These transition matrices control the process of amino acid identity changes, simulating protein sequence amino acid substitution dynamics. By integrating a state-of-the-art TCR–epitope binding prediction model as the backbone of our denoising neural network, we enable the network to predict the amino acid state changes at each diffusion step while explicitly learning the adaptive amino acid substitutions and functional constraints that govern epitope recognition. Ultimately, the model can be initiated from random amino acid sequences and generate new epitope-specific TCR sequences through a reverse denoising process, conditioned on target pMHC. To comprehensively evaluate DTCR’s performance, we conducted systematic experiments across 20 abundant epitopes in the dataset, all of which were completely unseen during the training phase of the model. Our results demonstrate that DTCR outperforms existing state-of-the-art TCR generation models in terms of specificity, biological conservation, and sequence diversity. *In silico* structural modeling and binding interface analyses further demonstrate that DTCR-generated TCRs form larger buried binding interfaces and more energetically favorable binding interactions with target epitopes in modeled TCR–pMHC complexes, supporting their high epitope-specific binding potential.

## Materials and methods

### Data collection

In this study, we focused on the TCR beta CDR3 which is the primary determinant of TCR binding specificity [3]. Regarding MHC sequences, we utilized pseudo-sequences containing residues crucial for epitope binding. To generate TCRs with high specificity for target pMHC complexes, the model requires a systematic collection of TCR–pMHC pairs from diverse public databases. We utilized the dataset from TCR-TRANSLATE [14], which aggregates data from three commonly used databases including VDJdb (https://vdjdb.cdr3.net/) [18], Immune Epitope Database (IEDB) (https://www.iedb.org/) [19], and McPAS-TCR, a manually curated catalogue of pathology‑associated T‑cell receptor sequences (http://friedmanlab.weizmann.ac.il/McPAS-TCR/) [20], and incorporates a large sample of partially labeled data from the Multiplex Identification of Antigen‑Specific T‑Cell Receptors (MIRA) [21] dataset. MIRA dataset can be accessed at https://clinicaltrials.gov (accession: NCT04494893). Since the MIRA dataset lacked specific MHC allele information, researchers employed MHCFlurry2.0 to infer the presenting MHC alleles based on individual haplotype information.

All data were aggregated into a unified schema. Ambiguous or non-standard Human Leukocyte Antigen (HLA) alleles and amino acid notations were standardized using mhcgnomes (https://github.com/pirl-unc/mhcgnomes) and tidytcells [22]; missing HLA alleles were computationally imputed using MHCflurry 2.0 [23]. Entries with missing or invalid HLA allele, peptide, or CDR3β information were discarded. Only human MHC Class I-restricted TCR–pMHC pairs associated with cluster of differentiation 8‑positive (CD8+) T cells were retained. A two-step de-duplication process was performed: completely duplicate TCR–epitope–MHC paired records were first removed, and near-duplicate (identical MHC alleles and *≥ 6- mer* peptide overlap) records were further filtered out to eliminate bias from highly homologous sequences. CDR3β sequences were filtered to *≤ 30* amino acids, and epitope peptides were restricted to 8–11 amino acids.

**Figure 2:**
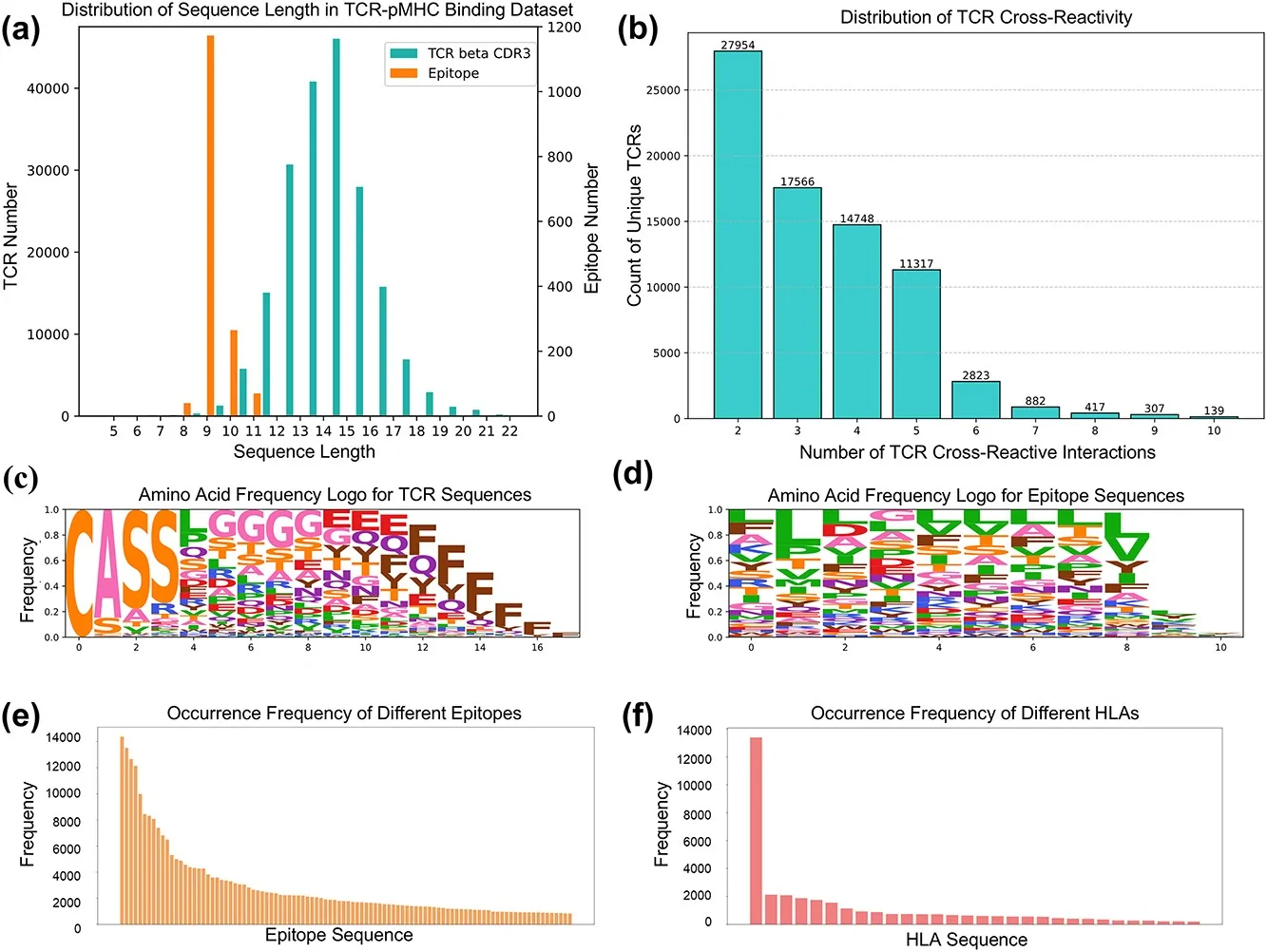
DTCR training and testing datasets; (a) distribution of sequence length in TCR–pMHC binding datasets; (b) the number of different epitopes recognized by cross-reactive TCRs that appear in both training and test sets; (c) amino acid frequency logo for TCR sequences; (d) amino acid frequency logo for epitope sequences; (e) occurrence frequency of different epitopes; (f) occurrence frequency of different HLAs.

To ensure a mutually exclusive train-test split, an epitope-exclusive dataset partition was performed. Due to the externally inferred MHC information, MIRA data was used exclusively for training. For the non-MIRA datasets, the top 20 most frequent pMHC pairs were reserved for testing, with the remaining data used for training. To mitigate data leakage, any training samples corresponding to these test-set epitopes paired with alternate MHC alleles were additionally removed. The 20 test set epitopes were thus completely excluded from the training set. The test set comprised ~68 K pairs, while the training set contained around 330 K pairs (1528 distinct peptide epitopes, 140 541 unique TCR sequences). Figure 2 provides details of the dataset.

### Diffusion model

Diffusion models are a class of generative deep learning models based on Markov chains [15], which reconstruct target data distributions through a forward adding noise process $q\left({x}_{1:T}|{x}_0\right)={\prod}_{t=1}^Tq\left({x}_t|{x}_{t-1}\right)$ and a backward denoising process ${p}_{\theta}\left({x}_{0:T}\right)=p\left({x}_T\right){\prod}_{t=1}^T{p}_{\theta}\left({x}_{t-1}|{x}_t\right)$. In the forward adding noise process, the model gradually transforms data into Gaussian noise, which is achieved through predefined Markov transition probabilities $q\left({x}_t|{x}_{t-1}\right)$. The data ${x}_0\sim q\left({x}_0\right)$ is then corrupted into a sequence of increasingly noisy latent variables ${x}_{1:T}={x}_1,{x}_2,\dots, {x}_T$. Ultimately, at time step T, ${x}_T$ becomes indistinguishable from random noise. In the backward denoising process, the learned parameterized denoising network ${p}_{\theta}\left({x}_{t-1}|{x}_t\right)$ attempts to reverse the above process, progressively reconstructing the original data from the noise. To train the parameterized denoising network, it is typical to optimize a variational upper bound on the negative log-likelihood:


(1)

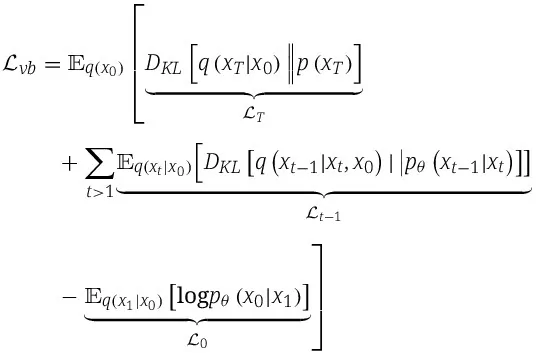



${\mathcal{L}}_T$ measures whether the corruption at timestep *T* reaches the stationary distribution and does not depend on *θ*. While ${\mathcal{L}}_{t-1}$ and ${\mathcal{L}}_0$ both depend on *θ*.

### Discrete diffusion framework of DTCR

The discrete diffusion model also needs to optimize a variational upper bound on the negative log-likelihood as formulation 1. It is necessary to define a proper $q\left({x}_t|{x}_{t-1}\right)$ for efficient training of ${p}_{\theta }$, because it enables effective sampling from $q\left({x}_t|{x}_0\right)$ and allows for the calculation of a tractable forward process posterior $q\left({x}_{t-1}|{x}_t,{x}_0\right)$ to compute Kullback–Leibler (KL) divergences necessary for optimization [24]. In the discrete domain, each element in ${x}_t$ can represent a discrete variable with *K* categories, where in TCR sequence data, *K* denotes the total number of distinct amino acids (tokens), allowing ${x}_t$ to be expressed as a stack of one-hot vectors. $q\left({x}_t|{x}_{t-1}\right)$ can be defined as follows:


(2) \begin{eqnarray*} q\left({x}_t|{x}_{t-1}\right)= Cat\left({x}_t;p={x}_{t-1}{Q}_t\right) \end{eqnarray*}

where $Cat\left(x;p\right)$ is a categorical distribution over the one-hot row vector *x* with probabilities given by the row vector *p.*  ${Q}_t$ is a transition matrix applied to each token in the TCR sequence independently, ${\left[{Q}_t\right]}_{i,j}=q\left({x}_t=j|{x}_{t-1}=i\right)$.

Following the original Discrete Denoising Diffusion Probabilistic Models (D3PM) [24], we can define the *t* step marginal distribution $q\left({x}_t|{x}_0\right)$ and *t* – 1 step posterior $q\left({x}_{t-1}|{x}_t,{x}_0\right)$:


(3) \begin{eqnarray*}q\left({x}_t|{x}_0\right)= Cat\left({x}_t;p={x}_0{\overline{Q}}_t\right)\end{eqnarray*}

(4) \begin{eqnarray*}q\left({x}_{t-1}|{x}_t,{x}_0\right) = & \frac{q\left({x}_{t-1}|{x}_0\right)q\left({x}_t|{x}_{t-1},{x}_0\right)}{q\left({x}_t|{x}_0\right)}\nonumber\\& = Cat\left({x}_{t-1};p=\frac{x_t{Q}_t^{\mathrm{T}}\odot{x}_0{\overline{Q}}_{t-1}}{x_0{\overline{Q}}_t{x}_t^{\mathrm{T}}}\right)\end{eqnarray*}

where ${\overline{Q}}_t={Q}_1{Q}_2\dots{Q}_t$*.*  *⊙* denotes element-wise product. ${\tilde{p}}_{\theta}\left({\tilde{x}}_0|{x}_t\right)$ is predicted by the denoising network and ${\tilde{x}}_0$ is the predicted distribution of ${x}_0$*.* Then it is combined with $q\left({x}_{t-1}|{x}_t,{x}_0\right)$to calculate ${p}_{\theta}\left({x}_{t-1}|{x}_t\right)$ in formulation 1.


(5) \begin{eqnarray*} {\displaystyle \begin{array}{c}{p}_{\theta}\left({x}_{t-1}|{x}_t\right)\propto \sum_{{\tilde{x}}_0}q\left({x}_{t-1},{x}_t|{\tilde{x}}_0\right){\tilde{p}}_{\theta}\left({\tilde{x}}_0|{x}_t\right)\end{array}} \end{eqnarray*}

Based on the above equations, we can start to train the denoising network. At each timestep *t,*  ${x}_t$ is sampled via $q\left({x}_t|{x}_0\right)$ for each residue in ${x}_0$ independently. And $q\left({x}_{t-1}|{x}_t,{x}_0\right)$ in ${\mathcal{L}}_{t-1}$ can be computed. ${\tilde{p}}_{\theta}\left({\tilde{x}}_0|{x}_t\right)$ is used to predict ${\tilde{x}}_0$*.* Then we can calculate ${p}_{\theta}\left({x}_{t-1}|{x}_t\right)$*.* It is worth noting that, even though the corruption and loss are calculated independently over each residue, the denoising network predicts ${\tilde{x}}_0$ based on the context of the entire TCR sequence.

We can then sample using the trained denoising network, beginning from the random noise ${x}_T$, where each residue in the sequence is randomly sampled from a uniform distribution over amino acids. Then ${x}_0$ can be sampled by iteratively sampling ${x}_{t-1}$ via ${p}_{\theta}\left({x}_{t-1}|{x}_t\right)$. It is worth noting that during both the training and sampling phases, the pMHC is employed as a conditioning factor, with no noise-addition or denoising processes applied to the pMHC sequences.

### Two kinds of corruption schemes simulate the T cell receptor amino acid substitution dynamics

Unlike the additive Gaussian noise in continuous diffusion models, discrete diffusion models can choose appropriate ${Q}_t$ to control data corruption and denoising processes, with the requirement that the rows of ${Q}_t$ must sum to 1, and the rows of ${\overline{Q}}_t={Q}_1{Q}_2\dots{Q}_t$ must converge to a known stationary distribution as *t* increases. By adding domain-dependent structures to ${Q}_t$, the quality of generated results can be improved and the model’s adaptability to specific tasks can be enhanced. Currently, there are several choices for ${Q}_t$. However, unlike discretized Gaussian and token embedding distance [24], TCR sequence amino acid substitutions are more dependent on evolutionary and functional constraints, rather than simple continuous space or semantic similarity. An absorbing state [24] in discrete diffusion refers to a fixed, irreversible terminal state that each token can only enter but not leave during the forward corruption process. In our DTCR with absorbing‑state masking corruption (DTCR-M) model, this absorbing state corresponds to a special [MASK] token: at each step, an amino acid either remains unchanged or is permanently converted to [MASK]. This simple masking scheme constrains the model to only erase information rather than simulate biologically meaningful amino acid substitutions, limiting its ability to generate novel and structurally valid TCR sequences. In contrast, random or BLOSUM-based transition matrices can better simulate the TCR sequence amino acid substitution dynamics.

#### Random-based transition matrix

Following Hoogeboom *et al*. [25], we used the transition matrix 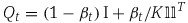, where the transition matrix ${Q}_t$ is doubly stochastic and uniform with a corruption schedule of ${\left(T-t+1\right)}^{-1}$. Information is linearly corrupted from time step 0 to time step *T*, with the stationary distribution being uniform, meaning that at time step *T*, the amino acid at each position of ${x}_T$ is randomly selected.

#### BLOSUM-based transition matrix

The BLOSUM62 matrix, derived from alignments of protein families in highly conserved regions, provides a scoring matrix based on amino acid substitution frequencies. Our BLOSUM corruption strategy is inspired by biologically informed discrete diffusion frameworks in protein sequence modeling, particularly the evolutionary-aware design proposed in EvoDiff [17]. Unlike uniform random corruption, this approach encodes natural evolutionary constraints into the forward diffusion process.

To convert BLOSUM62 scores into a valid transition matrix for discrete diffusion, we first applied a softmax operation over the raw BLOSUM substitution scores to convert them into non-negative probabilities. We then iteratively normalized rows and columns to enforce double stochasticity (both rows and columns sum to 1), which is a required property for Markov transition matrices in discrete diffusion models.

Compared with random and mask-based corruption, the BLOSUM-based scheme better captures biologically realistic amino acid substitution dynamics by prioritizing evolutionarily observed amino acid substitutions, yielding more biologically plausible TCR sequences. We chose an exponential scheduler that allows for smaller substitutions in smaller time steps and gradually increases substitution probability in larger time steps. By controlling the substitution probability to ~0.5 at T/2 through a carefully designed β-schedule, we can simulate the amino acid substitution dynamics in natural evolution.

### The denoising network simulates specificity screening process between T cell receptors and epitopes

The denoising network is used to predict ${\tilde{p}}_{\theta}\left({\tilde{x}}_0|{x}_t\right)$. In the original D3PM, a standard Transformer network can be employed to predict ${\tilde{x}}_0$. Understanding binding patterns between TCRs and epitopes is essential for TCR generation. Standard Transformer models have limited capability in accurately modeling the complex interaction mechanisms and biological constraints involved in TCR-epitope binding. However, by developing a denoising function based on a TCR-epitope binding prediction model, our model not only accurately captures the binding patterns between TCRs and epitopes but also simulates the specificity screening process between TCRs and epitopes. With ongoing advancements in TCR-epitope binding prediction models, our generative model will directly benefit, ensuring the model’s sustained effectiveness and innovative potential over the long term.

Based on the above ideas, we used the Physics-Inspired Sliding Transformer (PISTE) [26] as the base model for our denoising network. PISTE replaces traditional data-driven attention mechanisms with physics-driven dynamic processes, thereby intelligently navigating intricate biological sequence interaction landscapes. More details of PISTE are available in the Supplementary Notes (“Introduction of TCR-epitope Binding Prediction Model used for Constructing Denoising Network” section). We further designed a TCR sequence prediction module based on the above model. Since the original PISTE model used HLA pseudo-sequences as a skeleton and projected TCR and antigen onto HLA in the final alignment-based pooling module, which is misaligned with the downstream TCR sequence prediction task, we adjusted the module to project HLA and antigen onto TCR and obtain the corresponding TCR–pMHC representation. Subsequently, we designed a multi-layer self-attention module (Supplementary Notes “Training Details” section) to further process the representations obtained from the binding module and finally added a Multilayer Perceptron (MLP) layer to predict the TCR sequence, denoted as ${\tilde{x}}_0$. It is worth noting that the design of our denoising network is not limited to PISTE, and it can be adapted to use any advanced binding model as the base model by adjusting the binding model’s output.

## Results

### Experimental setup

Our model can utilize two types of transition matrices: a random-based matrix and a BLOSUM-based matrix. The BLOSUM-based matrix demonstrated superior performance in subsequent experiments. Therefore, we selected the model incorporating the BLOSUM-based transition matrix as our flagship model, termed DTCR. The model using the random-based transition matrix is referred to as DTCR with random‑based transition matrix (DTCR-R). The training details of DTCR and DTCR-R are in the Supplementary Notes (“Training Details” section). To further validate the performance improvements introduced by these transition matrices, we also designed a discrete diffusion model with an absorbing state, termed DTCR-M.

We selected two state-of-the-art TCR generation models for comparison: GRATCR [13], which is based on a pretrained TCR language model, and TCR-TRANSLATE [14], a translation-based model. Both follow a canonical two-stage paradigm: pretraining on large-scale unlabeled TCR sequences, followed by fine-tuning on labeled TCR–epitope or TCR–pMHC pairs. We used the official flagship models and public pretrained weights from GRATCR and TCR-TRANSLATE, and did not re-perform the pretraining step from scratch for either model. For GRATCR, we used the authors’ pretrained Epitope-BERT + TCR-GPT and fine-tuned it on the same training set using their default settings. For TCR-TRANSLATE, we used the authors’ pretrained TCRT5 and also fine-tuned it on our identical training set with their default hyperparameters. No extra hyperparameter tuning was performed during training; this choice avoids overfitting and maintains full reproducibility consistent with the original studies. Additionally, we included TCRs that bind to target pMHCs in the test set to assess the similarity between the generated TCRs and real binding TCRs.

Our test set comprises 20 abundant pMHCs from the collected data, with epitopes that have never appeared in the training set, a critical design choice that enables the model to be evaluated on novel epitopes excluded from the training process, thereby assessing its generalization capability. During the evaluation, each model generated 2000 TCRs for each pMHC, ultimately yielding 40 000 TCR–pMHC pairs. For autoregressive baselines GRATCR and TCR-TRANSLATE, we set beam search size to 2000, with all other inference hyperparameters at default. It is worth noting that GRATCR’s input only includes epitope sequences, so its generation results are TCR–epitope pairs. The evaluation metrics and two negative sampling strategies used in following experiments are detailed in the Supplementary Notes (“Evaluation Details” section).

To mitigate data leakage during binding specificity evaluation, the original training dataset was randomly split into 70% (~231 K pairs) for training all generative models (DTCR, DTCR-R, DTCR-M, GRATCR, and TCR-TRANSLATE), and the remaining 30% (~99 K pairs) was exclusively used for training the five independent binding prediction tools, with no overlapping pair between the two subsets. For all other analyses, all generative models were trained on the full training dataset (~330 K pairs, 1528 distinct epitopes). All models were trained and evaluated under identical data throughout the study to ensure unbiased comparison.

### DTCR can generate T cell receptors with high specificity

The training objective of DTCR is to generate highly specific TCRs for pMHCs. While wet-lab experiments can directly evaluate the specificity of generated TCRs, they are costly and impractical for large-scale assessments. In contrast, binding prediction tools can process vast amounts of data rapidly and provide reliable results. We employed five state-of-the-art binding prediction tools with diverse architectures to assess the specificity of generated TCRs, including ERGO [27], a peptide‑TCR matching prediction tool; TCR–Epitope Interaction Modelling (TEIM) [28]; PISTE [26]; NetTCR-2.0 [29], a convolutional neural network‑based TCR‑peptide binding prediction tool; and epiTCR [30], a Random Forest‑based TCR‑peptide binding predictor. These well-established tools have been adopted and assessed in a prior study [31] and are employed here to provide a preliminary computational assessment of the binding specificity of generated TCRs. The details of introduced five prediction tools are in the Supplementary Notes (“Introduction of TCR–epitope Binding Prediction Models for Evaluation” section). These predictors were trained on a held-out 30% subset of the original training data, which limits their performance but provides a conservative evaluation, while remaining independent of the 70% subset used to train all generative models to avoid data leakage and ensure unbiased evaluation. All performance evaluations were conducted on 20 unseen epitopes that were excluded from both generative model training and predictor training. Since the training dataset did not include experimentally validated negative TCR–pMHC pairs, we constructed artificial negative samples during evaluation using two distinct negative sampling strategies: the reference TCR negative sampling strategy and the unified epitope negative sampling strategy (Supplementary Notes “Evaluation Details” section). Area Under the Curve (AUC) values were calculated based on the binding scores of TCR–pMHC pairs: the positive pairs were composed of DTCR-generated TCR sequences paired with their corresponding target pMHC, while negative pairs were constructed using the exact same negative sampling pipeline implemented during the binding predictor training.

**Figure 3:**
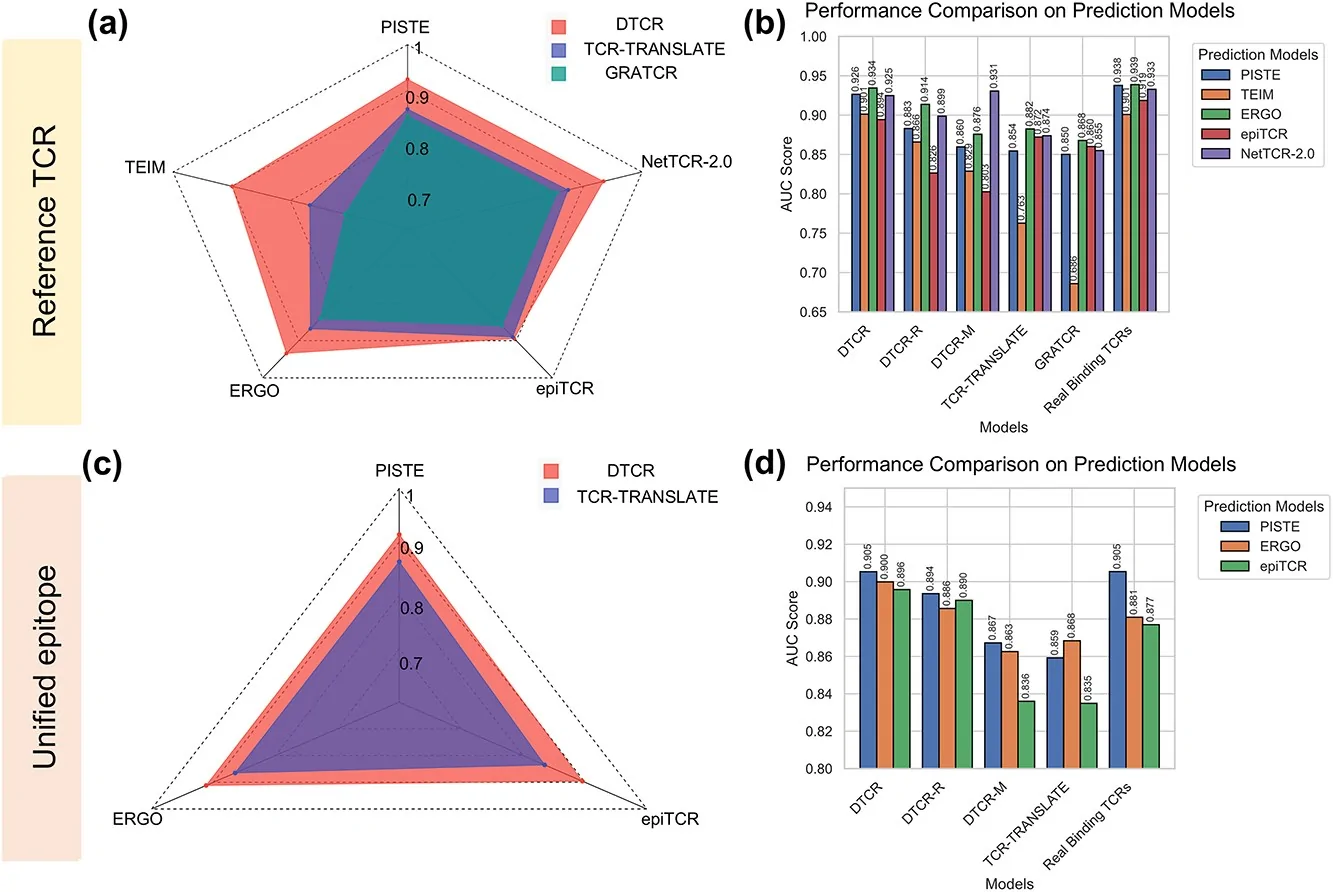
Evaluation of generated sequence binding specificity via distinct TCR–epitope binding prediction tools; (a) and (b) illustrate the performance of all models under the reference TCR negative sampling strategy; (c) and (d) show the performance of all models under the unified epitope negative sampling strategy.

We subsequently evaluated the specificity of TCRs generated by different models. Figure 3a and b illustrate the performance of all models under the reference TCR negative sampling strategy. According to Fig. 3b, DTCR achieves AUC values of 0.925 (PISTE), 0.899 (TEIM), 0.934 (ERGO), 0.894 (epiTCR), and 0.934 (NetTCR-2.0). These values are higher than those of the second-best comparative generation model TCR-TRANSLATE, by 7.56% (PISTE, 0.860), 17.21% (TEIM, 0.767), 7.60% (ERGO, 0.868), 0.45% (epiTCR, 0.890), 6.86% (NetTCR-2.0, 0.874). Figure 3c and d illustrate the performance of all models under the unified epitope negative sampling strategy. This strategy involves simultaneous sampling of epitopes and MHCs to pair with TCRs as negative pairs. TEIM and NetTCR-2.0, which cannot utilize MHC information, are incompatible with this strategy. Additionally, GRATCR, which generates only TCR–epitope pairs, cannot be fairly evaluated alongside other models under this strategy and is thus excluded. According to Fig. 3d, DTCR achieves AUC values of 0.914 (PISTE), 0.912 (ERGO), and 0.896 (epiTCR). These values are higher than those of TCR-TRANSLATE, by 5.91% (PISTE, 0.863), 5.43% (ERGO, 0.865), and 7.31% (epiTCR, 0.835).

Figure 3b and d also compare the performance of DTCR with DTCR-R and DTCR-M under the two negative sampling strategies. Under both strategies, AUC values achieved by DTCR are higher than those of DTCR-R and DTCR-M. Comparing the two negative sampling strategies, performances are generally higher under the reference TCR negative sampling strategy. This may be due to shortcut learning [32], where predictors tend to capture distributional differences between background TCRs and real TCRs, rather than the actual binding patterns of TCR–pMHC. However, DTCR achieved optimal performance under both strategies with minimal variation, further demonstrating its robustness. Importantly, our goal is not to provide a comprehensive benchmark of TCR–pMHC binding predictors. Instead, we used a fixed train–test split within our dataset and evaluated only on 20 held-out epitopes for the sole purpose of relative ranking between generative models. The high AUC values should therefore be interpreted as relative performance among generated TCRs, rather than as absolute or state-of-the-art performance of the binding predictors themselves.

To further examine the confounding impact of training-set TCR sequence similarity, we stratified experimentally validated binding TCRs by 95% sequence similarity (Lu *et al*. [31]; calculated via Cluster Database at High Identity with Tolerance (CD-HIT)) relative to training TCRs and compared their predictive performance across all evaluation pipelines (Supplementary Fig. S1a–h). Within our test setup consisting of 20 unseen epitopes, the stratified Receiver Operating Characteristic (ROC) results reveal no consistent AUC advantage for high-training-similarity TCRs under either negative sampling strategy, indicating that under this scenario, sequence overlap with training data does not lead to systematic, consistent overestimation of binding scores across the whole test set, even though subtle sequence-dependent biases may still exist within prediction models. We further quantified the fraction of high- and low-similarity sequences produced by each generative model via stacked bar charts (Supplementary Fig. S1i), which demonstrate that all DTCR variants generate fewer high-training-similarity TCRs than baseline models.

### DTCR-generated T cell receptors exhibit high consistency with natural T cell receptors in sequence similarity and generation probability distribution

Specific sequence patterns, which form the structural basis for potential biological activity, are conserved in natural TCRs; thus, comparing sequence features helps assess whether generated TCRs retain such evolutionarily constrained patterns. Similarly, validating the biological plausibility of generated sequences (i.e. their consistency with natural immune repertoire characteristics) provides evidence for their potential to adhere to *in vivo* immune diversification rules.

**Figure 4:**
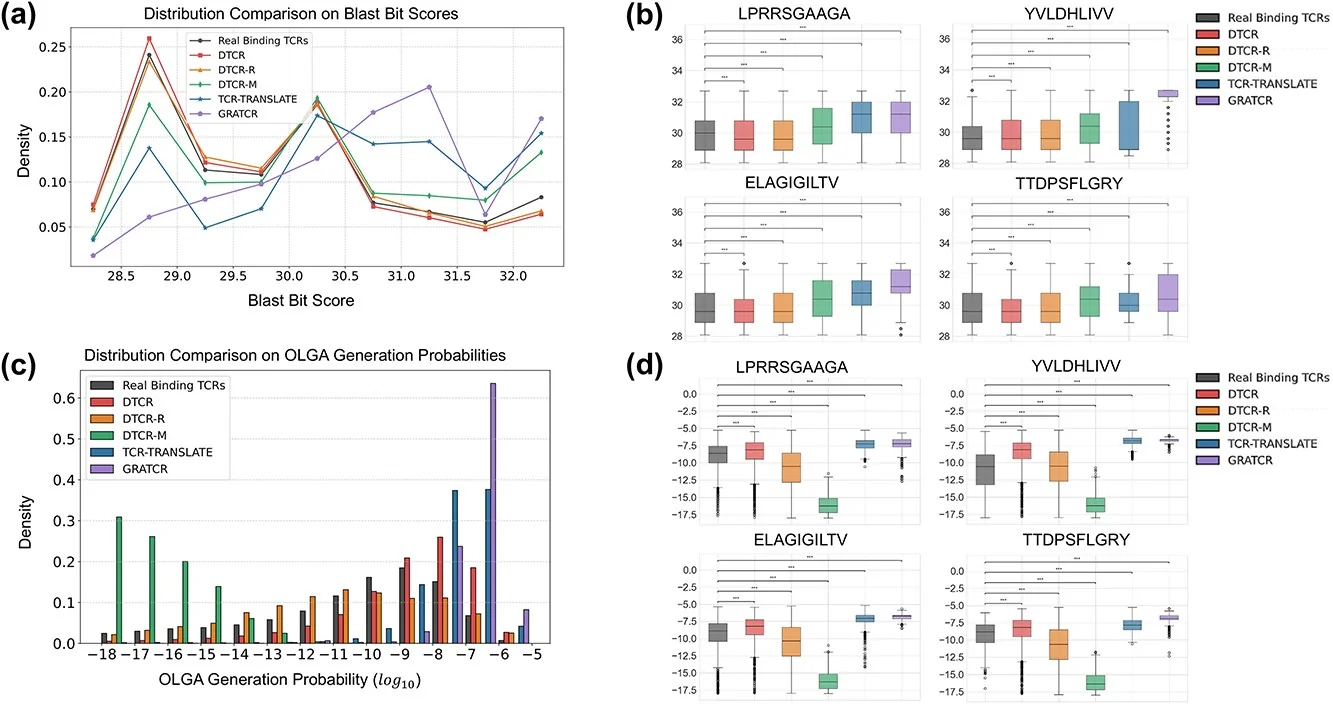
Assessment of the consistency between generated TCRs and real binding TCRs in BLAST bit score and generation probability distribution; (a) distribution comparison of BLAST bit scores for different models across all epitopes; (b) distribution comparison of BLAST bit scores for different models targeting specific epitopes (statistical significance was determined using the Mann–Whitney U test: ^*^, *P* < .05; ^**^, *P* < .01; ^***^, *P* < .001; ns, not significant); (c) distribution comparison of OLGA generation probabilities for different models across all epitopes; (d) distribution comparison of OLGA generation probabilities for different models targeting specific epitopes (statistical significance was determined using the Mann–Whitney U test: ^*^, *P* < .05; ^**^, *P* < .01; ^***^, *P* < .001; ns, not significant).

We used Basic Local Alignment Search Tool (BLAST) to align generated TCR amino acid sequences with a reference database containing 11 million unique TCR beta CDR3 sequences [33], with the BLOSUM62 matrix configured to quantify sequence homology. BLAST bit-scores, which reflect the strength of sequence similarity, show that DTCR, DTCR-R, and DTCR-M align with the homology patterns of real binding TCRs: over 90% of real binding TCRs have bit-scores between 28 and 33, and their generated sequences also concentrate in this interval (Fig. 4a). Notably, BLAST bit-scores measure sequence homology rather than functional binding capacity—public databases contain non-specific or anergic TCRs with high homology to functional ones, but this alignment indicates that DTCR-generated sequences retain the amino acid composition and length-dependent patterns typical of natural TCRs, providing a sequence-level foundation for potential biological relevance. In contrast, GRATCR and TCR-TRANSLATE produce sequences with higher bit-scores (Fig. 4a), deviating from the bimodal distribution (peaking at ~28.5 and 30) of real binding TCRs, suggesting they may overemphasize similarity at the cost of natural pattern recapitulation. Distribution across epitopes (Fig. 4b and Supplementary Fig. S2) further supports DTCR’s consistency with real TCR sequence features.

We also employed Optimized Likelihood estimate of immunoGlobulin Amino‑acid sequences (OLGA) [34], a tool that models V(D)J recombination to calculate the probability of a TCR sequence arising through natural immune diversification. OLGA probabilities reflect the likelihood of a sequence being generated *in vivo*, not its ability to bind epitopes. Real binding TCRs show the highest probabilities between ${10}^{-12}$ and ${10}^{-8}$, with symmetrically decreasing tails (Fig. 4c); DTCR and DTCR-R-generated sequences closely mirror this distribution, whereas GRATCR and TCR-TRANSLATE skew toward higher probabilities (${10}^{-7}$–${10}^{-6}$) and DTCR-M toward lower ones (Fig. 4c). This indicates that DTCR captures the probabilistic rules governing natural TCR generation, avoiding biologically implausible sequences that rarely emerge in real immune repertoires. Epitope-specific distributions (Fig. 4d and Supplementary Fig. S3) reinforce this consistency. Notably, the higher generation probabilities of GRATCR and TCR-TRANSLATE do not inherently indicate inferior performance. Such a pattern may reflect denser sampling within certain regions of sequence space, even though DTCR remains the most faithful to the natural distribution of real TCRs.

**Figure 5:**
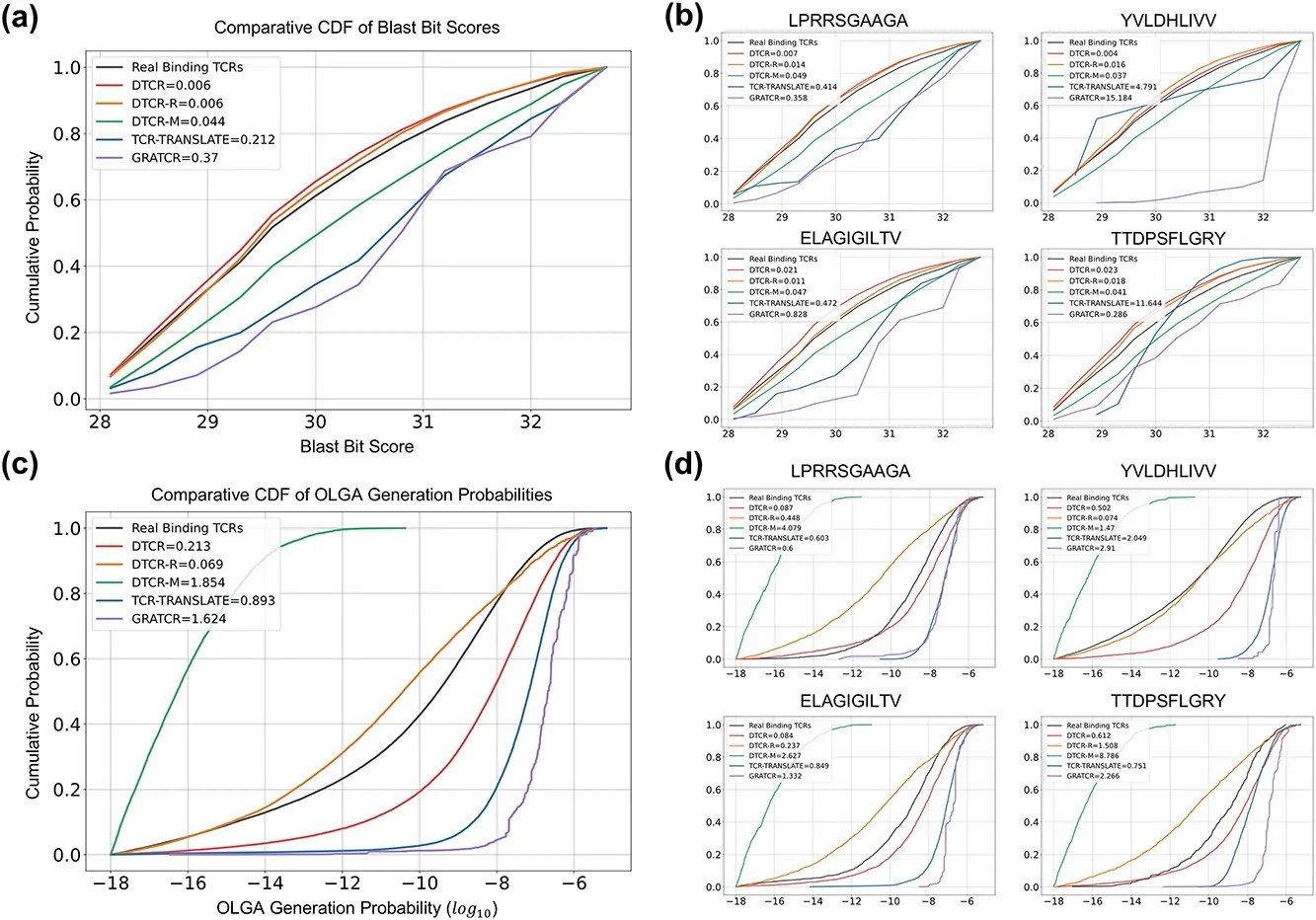
Cumulative distribution function (CDF) analysis of the consistency between generated TCRs and real binding TCRs; the KL divergence between generated TCRs and the real binding TCRs’ probability distributions is detailed in the accompanying legen; (a) CDF comparison of BLAST bit scores for different models across all epitopes; (b) CDF comparison of BLAST bit scores for different models targeting specific epitopes; (c) CDF comparison of OLGA generation probabilities for different models across all epitopes; (d) CDF comparison of OLGA generation probabilities for different models targeting specific epitopes.

KL divergence quantifies how closely generated sequences match the statistical distributions of real binding TCRs. For BLAST bit-scores (Fig. 5a), DTCR and DTCR-R exhibit minimal divergence (0.006), far lower than GRATCR (0.370) and TCR-TRANSLATE (0.212). For OLGA probabilities (Fig. 5c), DTCR-R (0.069) and DTCR (0.213) also outperform comparative models (GRATCR: 1.624; TCR-TRANSLATE: 0.893), confirming their superior recapitulation of natural sequence statistics. The lower divergence of DTCR-R in OLGA probabilities further suggests real TCR substitution dynamics involve randomness beyond BLOSUM matrix constraints. Notably, DTCR, DTCR-R, and DTCR-M consistently showed lower BLAST bit-score KL divergences than TCR-TRANSLATE and GRATCR across all epitopes (Supplementary Fig. S4). For OLGA probability KL divergences, DTCR and DTCR-R stably occupied the top two positions across nearly all epitopes (Supplementary Fig. S5), with only minor shifts in relative order observed among the remaining models, with a single minor exception seen for epitope YLQPRTFLL, where TCR-TRANSLATE achieved a lower value (0.144) than DTCR (0.270).

Together, these results demonstrate that DTCR-generated TCRs align with real binding TCRs in sequence homology and natural generation patterns—key indicators of biological plausibility. Importantly, these metrics do not equate to functional binding or immunogenicity; instead, they confirm generated sequences adhere to the sequence and natural generation rules of natural TCRs, laying groundwork for further computational and structural analyses (e.g. diversity and conservation analysis, and structural modeling of TCR–pMHC complexes).

### DTCR-generated T cell receptors exhibit stronger diversity and biological conservation

Diversity in TCR sequences is critical for covering potential binding patterns to a target epitope, though diversity alone does not guarantee functionality; instead, it reflects the capacity to explore biologically relevant sequence space under evolutionary constraints. Biological conservation, by contrast, ensures the preservation of key structural or functional motifs—such as evolutionarily conserved contact residues—that are essential for TCR folding and pMHC recognition. Together, these two features (diversity and conservation) provide evidence for the biological plausibility of generated sequences, laying a foundation for potential functionality (validated further through structural analyses).

To assess these properties, we used sequence logo plots to visualize position-specific amino acid conservation and diversity, and entropy values to quantify them (low entropy indicating high conservation, high entropy indicating high diversity). For the variable middle 14 positions, DTCR-generated sequences of length 16 showed entropy values highly consistent with real binding TCRs (Pearson correlation = 0.988). While the average entropy of DTCR-generated sequences (2.86 bits) was marginally higher than that of real TCRs (2.41 bits), this increase was concentrated in the core antigen-contact region (positions 5–12), where generated sequences consistently showed higher diversity than real binding TCRs, as shown in Supplementary Fig. S6 (length 16). Importantly, this higher entropy did not reflect randomness: positional entropy profiles aligned closely with real TCRs, indicating the diversity was guided by evolutionary constraints (e.g. avoiding destabilizing substitutions in structurally critical positions). Supplementary Fig. S6 extends this analysis to lengths 12–20 amino acids, confirming consistent conservation-diversity patterns across different sequence lengths.

**Figure 6:**
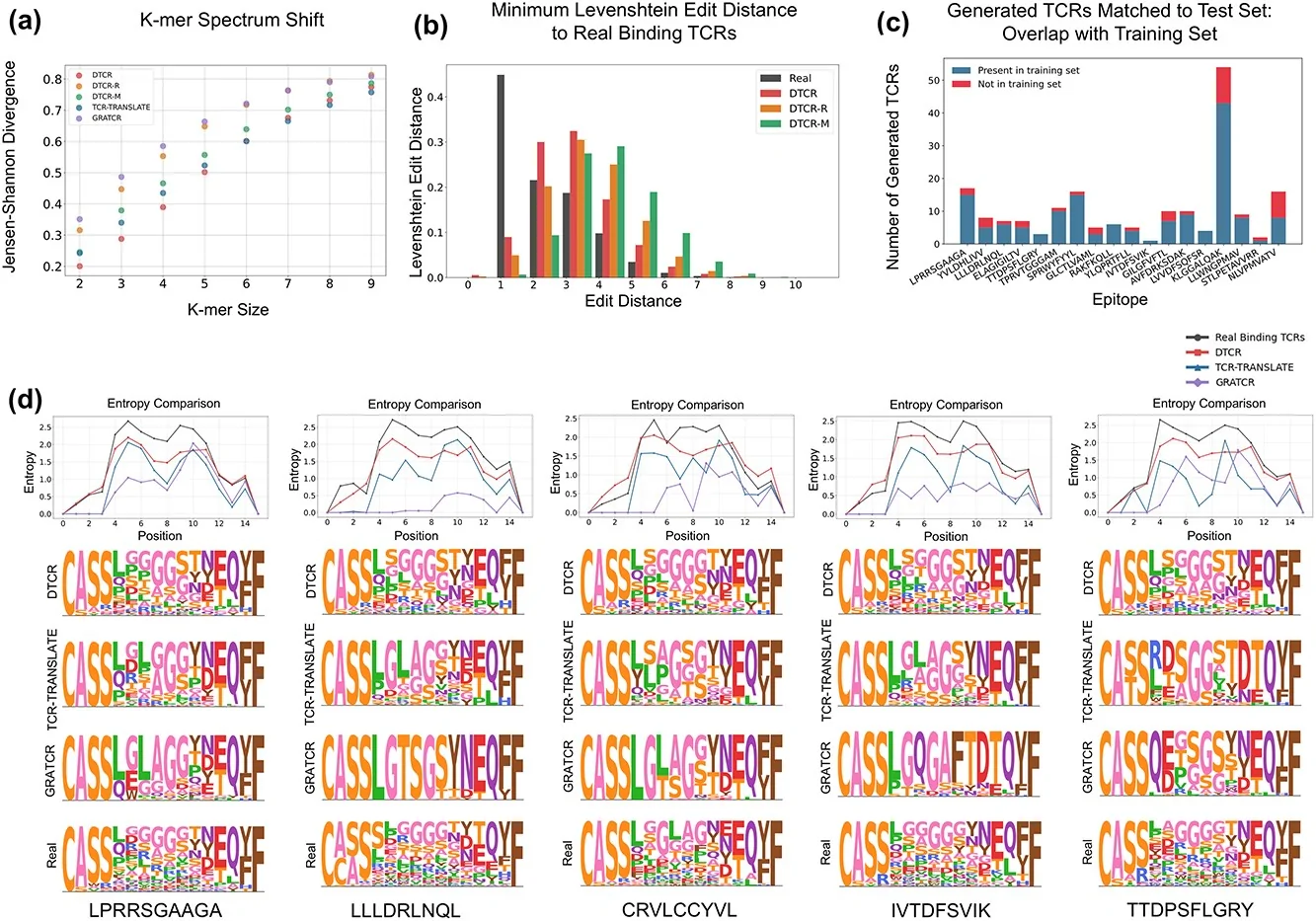
Assessment of the diversity and biological conservation of DTCR-generated TCRs; (a) the *k*-mer spectrum shift for different models; (b) minimum Levenshtein edit distance of generated TCRs to real binding TCRs; for real binding TCRs, edit distances were calculated between TCRs binding to a specific epitope and the remaining specific binding TCRs; (c) stacked counts of generated TCRs exactly matched the test set; lower segment: sequences also present in the training set (n = 153); upper segment: sequences unique to the test set and not observed in training (n = 38); (d) sequence logo plots for generated TCRs targeting different epitopes, with accompanying line plots indicating entropy values at each position.

Sequence logo plots and positional entropy analyses for five abundant epitopes (Fig. 6d) further demonstrated that DTCR-generated sequences maintained the natural balance of conservation and diversity: the middle antigen-contact positions (5–10) showed consistently high diversity (matching real TCRs), while flanking positions near the conserved C/F remained more constrained. In contrast, GRATCR and TCR-TRANSLATE exhibited less consistent positional entropy patterns compared with real binding TCRs, with stronger sequence conservation in parts of the central variable region. While these patterns differ from the natural conservation–diversity profile of functional TCRs, they may reflect a focused sampling strategy within a restricted sequence subspace, rather than inherent inferiority in model performance. Supplementary Fig. S7 validates this across all 20 test epitopes.

To assess local sequence features relevant to function (e.g. short functional motifs involved in pMHC binding), we used *k*-mer spectrum shift analysis [Jensen–Shannon (JS) divergence] to compare usage frequencies of short sequence segments (*k* = 2–8) between generated and real TCRs. As shown in Fig. 6a, DTCR showed the smallest JS divergence for *k* = 2–5 (0.200–0.502), indicating closer matches to real TCRs in short functional motifs (e.g. di/tripeptides involved in hydrogen bonding with epitopes). This outperformed DTCR-R (0.316–0.648), GRATCR (0.351–0.664), DTCR-M (0.246–0.557), and TCR-TRANSLATE (0.242–0.523) in this range. Notably, GRATCR exhibited the highest JS divergence across all *k* values while generating the least diverse TCR sequences. Its narrow sampling space tends to restrict recovery of the full repertoire of natural functional motifs, which produces substantial deviations in *k*-mer frequency distributions. For *k* >6, TCR-TRANSLATE showed slightly lower JS divergence, likely because it is better at learning longer-range dependencies in TCR sequences. Epitope-specific *k*-mer spectrum shift comparisons are provided in Supplementary Fig. S8.

Global sequence similarity (Levenshtein edit distance; Fig. 6b) confirmed that DTCR-generated sequences aligned more closely with real TCRs than DTCR-M, reflecting retention of overall sequence patterns. We further validated the generalization of DTCR by mapping generated TCRs against the training and test sets. As shown in Fig. 6c, among the generated TCRs that exactly matched the test set, 153 were present in the training set, while 38 were unique to the test set and absent from the training data, demonstrating that DTCR can discover novel natural binders beyond the training data.

To further evaluate the memorization effect of all models, we calculated the sequence overlap rate between generated TCRs and the training dataset, using natural TCRs from the held-out test set as the biological baseline. The natural test TCRs showed a baseline overlap rate of 0.12 with the training set, reflecting the inherent public clonotype sharing in human TCR repertoires. Among all generative models, DTCR achieved the lowest overlap rate of 0.22, which was closest to the natural baseline and lower than TCR-TRANSLATE (0.40) and GRATCR (0.65), indicating minimal memorization and strong generalization ability in DTCR.

Importantly, these metrics (entropy, *k*-mer usage, and edit distance) quantify sequence patterns rather than direct functionality; however, their consistency with real TCRs—particularly in conserved sequence motifs, position-specific diversity in antigen-contact regions, and short functional k-mers—supports the potential for these sequences to retain functional relevance, which is further validated by structural analyses in subsequent sections.

### Structural analysis of DTCR-generated T cell receptors

**Figure 7:**
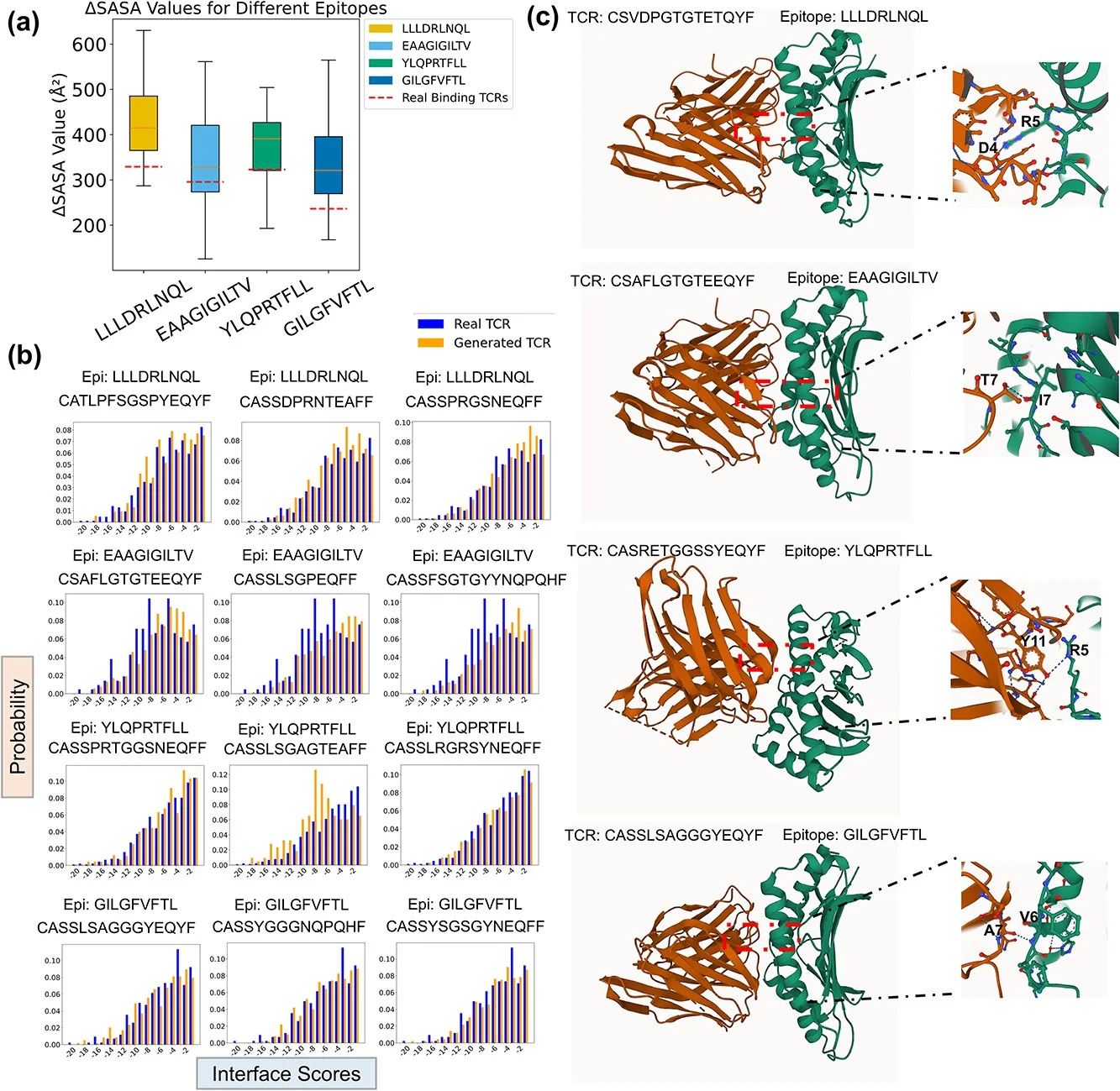
Structural analysis of DTCR-generated TCRs; (a) ΔSASA values for 100 generated TCRs across four epitopes; (b) distribution of binding interface scores between the generated TCRs and pMHC, calculated using RosettaDock-5.0; (c) visualization of the interactions between the generated TCRs and specific epitopes.

We further investigated whether the TCR sequences generated by DTCR exhibit high similarity to natural TCRs at structural level. We collected real pMHC–TCR complex Protein Data Bank (PDB) data from the Structural T‑cell Receptor Database (STCRDab) [35] for four different epitopes that are exclusively part of the test set and were not encountered by DTCR during training: LLLDRLNQL (PDB: 8dnt), EAAGIGILTV (PDB: 6tmo), GILGFVFTL (PDB: 1oga), and YLQPRTFLL (PDB: 7pbe). For each pMHC, we generated 100 TCRs and replaced the β CDR3 region in the original data. To obtain the structure of the TCR–pMHC complex after CDR3 replacement, we employed TCRdock [36], which provides an AlphaFold pipeline for predicting the structure of TCR:pMHC complexes. To evaluate whether the generated TCRs could form more stable structures, following the study of GRATCR [13], we calculated the change in solvent-accessible surface area (ΔSASA) of the TCR β CDR3 region before and after binding to the epitope. Figure 7a shows the ΔSASA values for 100 generated TCRs across the four epitopes. The red dashed lines represent the ΔSASA values of the real TCRs from the original data. A higher ΔSASA indicates a larger buried binding interface between TCR and epitope. For the four selected epitopes, the mean ΔSASA values of the TCRs generated by DTCR are as follows: 430.3 (LLLDRLNQL), 348.8 (EAAGIGILTV), 372.0 (YLQPRTFLL), and 333.9 (GILGFVFTL), which are higher than those of the real TCRs collected from STCRDab: 329.2 (LLLDRLNQL), 295.7 (EAAGIGILTV), 323.1 (YLQPRTFLL), and 236.4 (GILGFVFTL). For further comparison, the ΔSASA values of TCRs generated by GRATCR and TCR-TRANSLATE for these four epitopes are provided in Supplementary Fig. S9. DTCR exhibited a tendency toward higher ΔSASA values compared with GRATCR and TCR-TRANSLATE across all four epitopes. These results suggest that DTCR-generated TCRs form larger binding interfaces, which may contribute to improved binding stability. It should be noted that while ΔSASA quantifies the buried interface area, it does not correlate directly with binding affinity or specificity, necessitating experimental validation.

To further corroborate the above findings, we used RosettaDock-5.0 [37] to score the binding interface between the generated TCRs and pMHCs. The interface score is defined as the total score of the complex minus the total score of each partner in isolation [37]. Lower interface scores indicate more stable and favorable structural interface interactions. For each epitope, we selected three sequences from the 100 generated TCRs, with ΔSASA values falling within the mean ± 1 SD of the full dataset. As shown in the histogram in Fig. 7b, the generated TCRs exhibit similar or even lower interface scores compared to real TCRs on three epitopes. Specifically, for the epitopes LLLDRLNQL [generated TCRs: −4.66 (average), real TCR: −4.37 (average)] and YLQPRTFLL [generated TCRs: −5.05 (average), real TCR: −4.16 (average)], the TCRs generated by DTCR have lower interface scores. For the epitope GILGFVFTL, the interface scores of the generated TCRs are similar to those of the real TCR [generated TCRs: −4.15 (average), real TCR: −4.18 (average)]. However, for the epitope EAAGIGILTV, the interface scores of the generated TCRs are higher than those of the real TCR [generated TCRs: −4.59 (average), real TCR: −6.19 (average)]. Figure 7c and Supplementary Fig. S10 visualize the interactions between generated TCRs and specific epitopes. For instance, for the epitope LLLDRLNQL, the amino acid at the fourth position (D4) of the generated TCR beta CDR3 forms a hydrogen bond with the amino acid at the fifth position (R5) of the epitope.

## Discussion

This study addresses the critical challenge of generating epitope-specific TCR sequences with high binding specificity and biological plausibility. Traditional wet-lab experimental methods for TCR discovery are limited by inefficient and costly workflows. While significant advances have been made in computational epitope-specific TCR generation, existing frameworks are predominantly built on natural language modeling paradigms, with limited explicit integration of the evolutionary and functional constraints that shape naturally occurring, biologically active TCRs, and with insufficient utilization of the mechanistic insights into TCR–epitope binding captured by state-of-the-art prediction models. To address these limitations, we propose a novel TCR generation model based on discrete diffusion, named DTCR. This model leverages the inherent discrete nature of TCR sequences and integrates a state-of-the-art TCR–epitope binding prediction model to explicitly encode epitope-specific constraints. By simulating natural TCR amino acid substitution and specificity selection processes, DTCR enhances the binding specificity and sequence diversity of its generated TCRs.

Notably, the masking-based corruption strategy (DTCR-M) uses an absorbing-state scheme where tokens are kept unchanged or converted to a fixed [MASK] token. Although DTCR-M serves as a straightforward baseline for discrete diffusion models, it shows inferior performance relative to DTCR. Masking disrupts the local biochemical continuity of the sequence, and the fixed [MASK] token cannot encode or guide the model to learn biologically meaningful amino acid substitution patterns, thus limiting the model’s ability to exploit TCR–pMHC binding information. By comparison, DTCR-R, which uses a random corruption strategy, improves generation flexibility and better maintains sequence context during the diffusion process, yet still underperforms DTCR. This is because random corruption lacks explicit biological constraints. In contrast, DTCR with the BLOSUM-based corruption strategy most effectively captures natural amino acid substitution constraints and epitope-binding preferences, leading to higher binding specificity, sequence diversity, and biological plausibility.

Our analyses highlight several key observations regarding sequence generation behavior across different modeling paradigms. Within the discrete diffusion framework, DTCR incorporating BLOSUM-based substitution dynamics captures natural amino acid substitution preferences more closely, while DTCR-R demonstrates that randomness also contributes to realistic TCR generation profiles, indicating that native TCR sequence evolution likely involves both evolutionary constraints and stochasticity beyond fixed BLOSUM rules. Meanwhile, the elevated generation probabilities (computed via the OLGA tool, Fig. 4c) observed in GRATCR and TCR-TRANSLATE do not inherently reflect poorer performance, but may instead reflect denser sampling within restricted regions of TCR sequence space. DTCR stands out for its closer adherence to the natural statistical distribution of functional TCRs, as supported by consistent cumulative distribution function patterns and low divergence across epitopes. Together, these observations illustrate that integrating biologically informed corruption and binding guidance helps maintain the balance between diversity and conservation characteristic of natural repertoires. Computational structural analysis further suggests that such sequences can form stable, buried interfaces with target pMHC complexes. These observations remain computational in nature and serve only to support biological plausibility, not conclusive functional validation. Predicted binding scores reflect relative model behavior, and generalization limits of current TCR–epitope predictors on unseen epitopes still apply. Formal experimental validation is ultimately required to establish the functional and immunological relevance of these computationally generated sequences.

While the results demonstrate the utility of DTCR for epitope-specific TCR sequence generation, there remain several key opportunities to refine and extend the model for broader therapeutic applications.

First, our current BLOSUM-based corruption strategy adopts a position-agnostic assumption across CDR3 positions to ensure model simplicity and training stability. Although different CDR3 positions serve distinct structural and functional roles in antigen recognition, estimating reliable position-specific transition matrices remains challenging due to variable sequence lengths and limited data for rare CDR3 lengths. Our approach provides a robust baseline, and future work could explore reliable position-aware and length-aware corruption strategies to better model biologically realistic amino acid substitution dynamics.

Second, the TCR–pMHC binding predictors used for specificity evaluation have inherent limitations, and their generalization performance on unseen epitopes remains a major open challenge in immunology [31]. As quantified in Supplementary Fig. S1 via stratification at the 95% sequence similarity cutoff, high-training-similar TCR subsets exhibit no sustained AUC advantage across all test configurations, and all DTCR variants generate fewer high-training-similar TCRs relative to the baselines. Even with these analyses, computational evaluation still carries inherent uncertainty under the current state of the field. Our analysis cannot fully disentangle genuine epitope-binding signals from spurious sequence-matching biases embedded within binding prediction tools. It remains plausible that such predictors only reliably capture functional TCR–pMHC interactions for sequences similar to training data, and no negative sampling pipeline can completely eliminate this confounding bias. Accordingly, all cross-model AUC comparisons are interpreted merely as preliminary relative rankings, rather than conclusive evidence of genuine epitope-specific recognition capacity. More robust validation of binding specificity and affinity will require advanced structure-aware modeling tools and orthogonal wet-lab experiments.

Third, we adopted a strict epitope-level train-test split, with no overlapping epitopes between the training and test sets. However, this strategy cannot eliminate TCR sequence overlap between datasets, as some TCRs are inherently cross-reactive and capable of recognizing multiple distinct epitopes. As our model learns the intrinsic sequence patterns of natural TCR repertoires, the TCR sequences generated by DTCR may also exhibit such cross-reactive properties. This highlights the need for refined cross-reactivity profiling and functional experimental validation of generated TCRs in real immune contexts to confirm their clinical utility and safety.

Fourth, relying on a single structural modeling tool for TCR–pMHC complexes (TCRdock) may incompletely cover the conformational landscape, reducing the reliability of our *in silico* structural analysis. Integrating multiple structure prediction pipelines could improve the robustness of structural evaluation in future work.

Finally, the current model focuses only on TCR β CDR3 generation, whereas natural TCRs function as paired αβ heterodimers. Therefore, modeling paired TCR αβ chains remains an essential direction for improving the biological realism and therapeutic relevance of generated TCRs.

**Key Points**

- DTCR represents a novel discrete diffusion framework dedicated to epitope-specific T cell receptor (TCR) sequence generation, which simulates natural amino acid substitution via a Blocks Substitution Matrix (BLOSUM)-based transition matrix and incorporates a TCR–epitope binding prediction module to support controllable and biologically plausible generation.
- DTCR yields improved predicted binding specificity across five mainstream evaluation tools, with relative Area Under the Curve (AUC) gains of 0.45%–17.21% over the comparative method TCR-TRANSLATE, and maintains robust performance under two distinct negative sampling strategies.
- TCRs generated by DTCR closely recapitulate key characteristics of natural immune repertoires, including sequence homology [Kullback–Leibler (KL) divergence = 0.006], Optimized Likelihood estimate of immunoGlobulin Amino‑acid sequences (OLGA)-derived generation probabilities (KL divergence = 0.213), and positional diversity–conservation balance (Pearson correlation = 0.988).
- *In silico* structural analysis reveals that DTCR-generated TCRs form larger buried binding interfaces (Δ solvent-accessible surface area) and more stable interactions with peptide-major histocompatibility complexes, providing computational support for its potential value in immunotherapy development.

## Supplementary Material

Supplementary_Material_bbag438

## Data Availability

DTCR was implemented in Python and can be easily installed via PyPI. Its source code is available at https://github.com/skybluewhy/DTCR.
