# Supplementary material for: DTCR: generating realistic, diverse, and epitope-specific T cell receptor sequences via a discrete diffusion model
Source: Brief Bioinform. 2026 Aug 27;27(4):bbag438. doi: 10.1093/bib/bbag438 (PMC13518066; doi:10.1093/bib/bbag438)
Supplement: Supplementary file 1 [file supplementary_material_bbag438.docx]

**DTCR: Generating Realistic, Diverse, and Epitope-Specific TCR Sequences via a Discrete Diffusion Model**

**Supplementary Materials**

Haoyan Wang^1^, Tianyi Zang^1,*^, and Yadong Liu^1,2,*^

^1^Faculty of Computing, Harbin Institute of Technology, Harbin, Heilongjiang, 150001, China.

^2^Zhengzhou Research Institute, Harbin Institute of Technology, Zhengzhou, Henan, 450000, China.

^*^Corresponding authors: Tianyi Zang: [tianyi.zang@hit.edu.cn](mailto:tianyi.zang@hit.edu.cn), Yadong Liu: [ydliu@hit.edu.cn](mailto:ydliu@hit.edu.cn)

Content

[Figure S1. ROC analysis stratified by TCR sequence similarity to training data and distribution of training-set similarity across generated TCRs	3](#_Toc169521455)

[Figure S2. Distribution of BLAST Bit Scores for Generated TCRs Across Different Epitopes	4](#_Toc169521455)

[Figure S3. Distribution of OLGA Generation Probabilities for Generated TCRs Across Different Epitopes	5](#_Toc169521456)

[Figure S4. Comparative Cumulative Distribution Function (CDF) of BLAST Bit Scores for Generated TCRs Across Different Epitopes	6](#_Toc169521457)

[Figure S5. Comparative Cumulative Distribution Function (CDF) of OLGA Generation Probabilities for Generated TCRs Across Different Epitopes	7](#_Toc169521458)

[Figure S6. Entropy at Different Positions in DTCR-Generated TCRs and Real Binding TCRs with Different Lengths	8](#_Toc169521459)

[Figure S7. Comparative Entropy Values at Each Position of Generated TCRs with Length 16 Targeting Different Epitopes	9](#_Toc169521460)

[Figure S8. The K-Mer Spectrum Shift (Generated TCRs vs. Real TCRs) for Different Models Across Different Epitopes	10](#_Toc169521461)

[Figure S9. ΔSASA Distributions (Generated TCRs) for Different Models Across Different Epitopes	11](#_Toc169521461)

[Figure S10. Visualization of the Interactions Between Generated TCRs and Specific Epitopes	12](#_Toc169521461)

[Supplementary Notes	14](#_Toc169521473)

[1.	Introduction of TCR-epitope Binding Prediction Model used for Constructing Denoising Network	14](#_Toc169521474)

[2.	Training Details	15](#_Toc169521475)

[3.	Evaluation Details	15](#_Toc169521475)

[4.	Introduction of TCR-epitope Binding Prediction Models for Evaluation	19](#_Toc169521476)

[References	20](#_Toc169521483)

**Figure S1. ROC analysis stratified by TCR sequence similarity to training data and distribution of training-set similarity across generated TCRs.** (a–e) ROC curves of experimentally validated real binding TCRs, stratified by sequence similarity relative to training TCRs (high similarity: ≥95%; low similarity: <95%, calculated via CD-HIT), assessed under reference TCR negative sampling using five independent TCR specificity prediction tools. (f–h) Corresponding stratified ROC curves under unified epitope negative sampling for three prediction tools. Across all evaluation configurations with 20 unseen epitopes, TCRs sharing high sequence similarity with the training set show no consistent AUC advantage over low-similarity counterparts. (i) Stacked bar chart quantifying the proportional fractions of TCRs with high and low training-set similarity from each generative model, as well as real binding TCRs. All DTCR variants generate TCRs dominated by low training-similarity sequences, with fewer high-similarity TCRs compared to baseline generative models.

**Figure S2. Distribution of BLAST Bit Scores for Generated TCRs Across Different Epitopes**. The corresponding epitopes are labeled above the box plots (Statistical significance was determined using the Mann‑Whitney U test: *, P < 0.05; **, P < 0.01; ***, P < 0.001; ns, not significant).

**Figure S3. Distribution of OLGA Generation Probabilities for Generated TCRs Across Different Epitopes**. The corresponding epitopes are labeled above the box plots (Statistical significance was determined using the Mann‑Whitney U test: *, P < 0.05; **, P < 0.01; ***, P < 0.001; ns, not significant).

**Figure S4. Comparative Cumulative Distribution Function (CDF) of BLAST Bit Scores for Generated TCRs Across Different Epitopes.** The Kullback-Leibler (KL) divergence between generated TCRs and the real binding TCRs' probability distributions is detailed in the accompanying legend. The corresponding epitopes are labeled above the line charts.

**Figure S5. Comparative Cumulative Distribution Function (CDF) of OLGA Generation Probabilities for Generated TCRs Across Different Epitopes.** The Kullback-Leibler (KL) divergence between generated TCRs and the real binding TCRs' probability distributions is detailed in the accompanying legend. The corresponding epitopes are labeled above the line charts.

**Figure S6. Entropy at Different Positions in DTCR-Generated TCRs and Real Binding TCRs with Different Lengths.** The corresponding lengths are labeled above the bar charts.

**Figure S7. Comparative Entropy Values at Each Position of Generated TCRs with Length 16 Targeting Different Epitopes.** The corresponding epitopes are labeled above the line charts.

**Figure S8. The K-Mer Spectrum Shift (Generated TCRs vs. Real TCRs) for Different Models Across Different Epitopes**. The corresponding epitopes are labeled above the line charts.

**Figure S9. ΔSASA Distributions (Generated TCRs) for Different Models Across Different Epitopes.** The corresponding epitopes are labeled above the box plots.

**Figure S10. Visualization of the Interactions Between Generated TCRs and Specific Epitopes.** For TCR CASRDGRNSNQPQHF and epitope LLLDRLNQL, the amino acid at the seventh position (R7) of the generated TCR beta CDR3 forms a hydrogen bond with the amino acid at the eighth position (Q8) of the epitope. For TCR CAITGRRGNTEAFF and epitope LLLDRLNQL, the amino acid at the fourth position (T4) of the generated TCR beta CDR3 forms a hydrogen bond with the amino acid at the fifth position (R5) of the epitope. For TCR CSSQREGSYNEQFF and epitope EAAGIGILTV, the amino acid at the sixth position (E6) of the generated TCR beta CDR3 forms a hydrogen bond with the amino acid at the ninth position (T9) of the epitope. For TCR CASSGTGFTETQYF and epitope EAAGIGILTV, the amino acid at the ninth position (T9) of the generated TCR beta CDR3 forms a hydrogen bond with the amino acid at the sixth position (G6) of the epitope. For TCR CSAPLAGARSYEQYF and epitope GILGFVFTL, the amino acid at the ninth position (R9) of the generated TCR beta CDR3 forms a hydrogen bond with the amino acid at the sixth position (V6) of the epitope. For TCR CASSFFPTGAGETQYF and epitope GILGFVFTL, the amino acid at the seventh position (P7) of the generated TCR beta CDR3 forms a hydrogen bond with the amino acid at the sixth position (V6) of the epitope. For TCR CASSLRGRSYNEQFF and epitope YLQPRTFLL, the amino acid at the sixth position (S9) of the generated TCR beta CDR3 forms a hydrogen bond with the amino acid at the one hundred and fifty-fifth position (Q155) of the HLA. And Q155 of the HLA forms a hydrogen bond with the amino acid at the fifth position (R5) of the epitope. For TCR CASSSLLAGSYNEQFF and epitope YLQPRTFLL, the amino acid at the tenth position (S10) of the generated TCR beta CDR3 forms a hydrogen bond with the amino acid at the one hundred and fifty-fifth position (Q155) of the HLA. And Q155 of the HLA forms a hydrogen bond with the amino acid at the fifth position (R5) of the epitope.

Supplementary Notes

1. Introduction of TCR-epitope Binding Prediction Model used for Constructing Denoising Network

We used the Physics-Inspired Sliding Transformer (PISTE) [1] as the base model for our denoising network. We first briefly introduce this model and then explain the design of our denoising network. PISTE replaces traditional data-driven attention mechanisms with physics-driven dynamic processes, guiding amino acid residues to adjust their positions along interaction gradient fields, thereby intelligently navigating intricate biological sequence interaction landscapes. This approach improves the accuracy of TCR-antigen-HLA binding predictions and demonstrates robust generalization capabilities for rare sequences. The PISTE model mainly includes the following modules: (1). Sequence Encoder Module: Using one-dimensional convolutions to extract subsequence features from TCR, antigen, and HLA sequences. (2). Sliding Attention Module: Sliding attention is a physics-inspired dynamic process that infers interactions between residues based on their spatial proximity and featural correlations. It then finds patterns through an iterative process, progressively "dragging" residues from one sequence towards residues in another sequence based on the magnitude of residue interactions (attention). (3). Alignment-based Pooling Module: Proposes a systematic method to transform variable-length biological sequences into fixed-length sequences by using HLA pseudo-sequences as a skeleton, projecting TCR and antigen sequences onto it, and converting them into fixed-length sequences.

Next, we designed a TCR sequence prediction module based on the above model. Since the original PISTE model used HLA pseudo-sequences as a skeleton and projected TCR and antigen onto HLA in the final alignment-based pooling module, which is misaligned with the downstream TCR sequence prediction task, we adjusted the module to project HLA and antigen onto TCR and obtain the corresponding TCR-pMHC representation. Subsequently, we designed a multi-layer self-attention module to further process the representations obtained from the binding module and finally added a MLP layer to predict the TCR sequence, denoted as $\tilde{x}_{0}$. It is worth noting that the design of our denoise network is not limited to PISTE, and it can be adapted to use any advanced binding model as the base model by adjusting the binding model's output.

2. Training Details

The denoising networks of DTCR and its variants (DTCR-R and DTCR-M) share the same basic architecture. Specifically, each denoising network consists of a multi-layer self-attention (MSA) module and a MLP layer. The MSA module is implemented as a stack of 3 BERT encoder layers with 8 attention heads, a hidden size of 256, and an intermediate feed-forward size of 512. This module employs multi-head self-attention to capture long-range sequence dependencies, alongside layer normalization and feed-forward networks for stable representation learning. The PISTE model was pre-trained on the same training set as our generative model using the unified epitope strategy for negative sampling, and its parameters were frozen during generative model training to retain its well-learned effective representations. For DTCR-M, valid representations cannot be extracted from PISTE; we directly concatenate TCR-pMHC sequences and extract features from neural network embeddings instead. The timestep of all three models is set to 128. We trained DTCR, DTCR-R, and DTCR-M using the AdamW optimizer for 30 epochs with a learning rate of 1e-4, a dropout probability of 0.1, and a batch size of 128. All experiments were conducted on an NVIDIA GeForce RTX 4090.

3. Evaluation Metrics

**AUC**

The AUROC is defined as the area under the Receiver Operating Characteristic (ROC) curve, which plots the True Positive Rate (TPR) against the False Positive Rate (FPR) across different decision thresholds. The TPR, also known as sensitivity or recall, is calculated as:

$$\begin{aligned} TPR=\frac{True Positives}{True Positives+False Negatives} \#\left（ 1 \right） \end{aligned}$$

The FPR is calculated as:

$$\begin{aligned} FPR=\frac{False Positives}{False Positives+True Negatives} \#\left( 2 \right) \end{aligned}$$

We used AUROC to evaluate the specificity of generated TCRs in section: DTCR can generate TCRs with high specificity.

**Cumulative distribution functions (CDF)**

Cumulative distribution functions (CDF) represent the probability that a random variable takes on a value less than or equal to a specific value. The CDF of a random variable X is defined as:

$$\begin{aligned} F\left( x \right)=P\left( X\leq x \right)\#\left( 3 \right) \end{aligned}$$

We calculated the cumulative distribution function (CDF) curves for each model on BLAST bit scores and OLGA generation probabilities in section: DTCR-Generated TCRs exhibits high consistency with natural TCRs in sequence similarity and generation probability distribution.

**Entropy**

Entropy quantifies the amount of information produced by a stochastic source of data. For a discrete random variable X, the entropy H(X) is defined as:

$$\begin{aligned} H\left( X \right)=-\sum_{i=1}^{n} P\left( x_{i} \right){log}_{b}P\left( x_{i} \right) \#\left( 4 \right) \end{aligned}$$

$P(x_{i})$ represents the probability of the i-th outcome of the random variable X. And b is the base of the logarithm, which determines the unit of entropy (base 2 for bits, base e for nats, and base 10 for dits). We calculated entropy to evaluate the diversity and conservation of generated TCR sequences at each position in section: DTCR-Generated TCRs exhibit stronger diversity and biological conservation.

**KL divergence**

KL divergence quantifies the difference between two probability distributions over the same variable. The KL divergence for continuous distribution P and Q is defined as:

$$\begin{aligned} D_{KL}(P|\left| Q \right)=\int_{-\infty}^{\infty} p\left( x \right)log\frac{p\left( x \right)}{q\left( x \right)}dx \#\left( 5 \right) \end{aligned}$$

where p(x) and q(x) are the probability density functions of P and Q, respectively. We calculated the Kullback-Leibler (KL) divergence between generated TCRs and the real binding TCRs' probability distributions on BLAST bit scores and OLGA generation probabilities in section: DTCR-Generated TCRs exhibit high consistency with natural TCRs in sequence similarity and generation probability distribution.

**JS divergence**

Jensen-Shannon (JS) divergence can measure the similarity between two probability distributions. Unlike Kullback-Leibler (KL) divergence, which is asymmetric, JS divergence is symmetric, providing a more balanced view of the divergence between distributions. For two probability distributions P and Q, the JS divergence is defined as:

$$\begin{aligned} D_{JS}(P||Q)=\frac{1}{2}D_{KL}(P||M)+\frac{1}{2}D_{KL}(Q||M) \#\left( 6 \right) \end{aligned}$$

where M is the average of the two distributions, defined as:

$$\begin{aligned} M=\frac{1}{2}\left( P+Q \right)\#\left( 7 \right) \end{aligned}$$

We calculated the Jensen-Shannon (JS) divergence of k-mer usage frequency distributions at different k values between generated and real TCRs in section: DTCR-Generated TCRs exhibit stronger diversity and biological conservation.

**Pearson correlation coefficient**

The Pearson correlation coefficient (r) is a measure of the strength and direction of the linear relationship between two continuous variables. It is defined as the covariance between the two variables divided by the product of their standard deviations:

$$\begin{aligned} r=\frac{Cov\left( X,Y \right)}{\sigma_{X}\sigma_{Y}}=\frac{\sum_{i=1}^{n} \left( X_{i}-\bar{X} \right)\left( Y_{i}-\bar{Y} \right)}{\sqrt{\sum_{i=1}^{n} \left( X_{i}-\bar{X} \right)^{2}}\cdot\sqrt{\sum_{i=1}^{n} \left( Y_{i}-\bar{Y} \right)^{2}}} \#\left( 8 \right) \end{aligned}$$

This formula quantifies the degree to which two variables linearly co-vary, with values ranging from -1 (perfect negative linear relationship) to 1 (perfect positive linear relationship), and 0 indicating no linear relationship. We computed the Pearson correlation coefficient between the entropy values of the generated TCRs and those of the real TCRs at each position in section: DTCR-Generated TCRs exhibit stronger diversity and biological conservation.

**Two negative sampling strategies in specificity evaluation**

To assess the specificity of the generated TCR, we conducted comprehensive evaluations using multiple TCR-epitope binding prediction tools. As negative sampling strategies may affect model evaluation [2], we adopted two widely used approaches to reduce sampling-induced variability [1]. In the reference TCR strategy, for each positive TCR–pMHC pair, we constructed a negative pair by pairing the same pMHC with a TCR randomly sampled from a library of unannotated CDR3β sequences. Strict filters were applied: only full-length CDR3β sequences free of non-standard amino acids were retained, and any sequences annotated as positive binders in our training and test sets were strictly excluded. In the unified epitope strategy, negative pairs were generated by pairing each positive TCR with an epitope or MHC sampled according to its frequency distribution in the dataset. In both schemes, negative pairs were guaranteed not to overlap with positive pairs, and the number of negative samples was matched to that of positive binding pairs. For reference TCR library, we collected TCR repertoire data from 85 healthy individuals from a large-scale high-throughput immune sequencing study conducted by Dean et al. [3]. After removing duplicate sequences and those containing wildcards (such as '*' or 'X'), we obtained over 11 million unique TCR beta CDR3 sequences, which served as our reference TCR library.

4. Introduction of TCR-epitope Binding Prediction Models for Evaluation

With the advancement of high-throughput sequencing technologies, databases such as VDJdb [4], IEDB [5], and McPAS-TCR [6] have accumulated extensive TCR and epitope binding data. Machine learning and deep learning methods can rapidly capture potential patterns of TCR-epitope interactions from these large-scale datasets. Researchers have developed various prediction approaches, including specificity predictions based solely on TCR sequences [7], binding predictions for specific epitope [8], and recently, universal prediction models applicable to broad ranges of TCR-epitope pairs [1,9–12]. We employed five state-of-the-art binding prediction tools with diverse architectures to assess the specificity of generated TCRs, including ERGO [9], TEIM [10], PISTE [1], NetTCR-2.0 [11], and epiTCR [12], which were proposed to estimate TCR–epitope binding affinity across a broad range of epitopes similar to those present in training datasets, yet exhibit limited generalization capacity toward fully unseen epitopes.

ERGO employs long short-term memory (LSTM) and pre-trained autoencoders to encode TCR and epitope sequences, integrating these with a multilayer perceptron (MLP) to predict TCR-epitope binding [9].

TEIM conducts pre-training at the sequence level, followed by fine-tuning at the residue level, using few-shot learning strategies to accurately predict TCR-epitope binding, as well as residue distances and contact sites [10].

PISTE combines biological and physical prior knowledge, leveraging a sliding attention mechanism to predict binding probabilities between TCRs and peptide-human leukocyte antigen (HLA) complexes [1].

NetTCR-2.0 encodes CDR3 and peptide sequences using the BLOSUM50 matrix. It then uses a 1D convolutional layer and a max-pooling layer to extract features from these encoded sequences. The extracted features are then concatenated and fed into a dense layer to predict the binding probability [11].

epiTCR processes TCR CDR3b and peptide sequences by encoding them with the BLOSUM62 matrix and then flattening the matrices into vectors. These vectors are concatenated and used as input features for the Random Forest classifier, which predicts the binding probability between TCRs and peptides [12].

References

1. Feng Z, Chen J, Hai Y, et al. Sliding-attention transformer neural architecture for predicting T cell receptor–antigen–human leucocyte antigen binding. Nat. Mach. Intell. 2024; 6:1216–1230

2. Jiang Y, Huo M, Cheng Li S. TEINet: a deep learning framework for prediction of TCR–epitope binding specificity. Brief. Bioinform. 2023; 24:bbad086

3. Dean J, Emerson RO, Vignali M, et al. Annotation of pseudogenic gene segments by massively parallel sequencing of rearranged lymphocyte receptor loci. Genome Med. 2015; 7:123

4. Shugay M, Bagaev DV, Zvyagin IV, et al. VDJdb: a curated database of T-cell receptor sequences with known antigen specificity. Nucleic Acids Res. 2018; 46:D419–D427

5. Vita R, Mahajan S, Overton JA, et al. The Immune Epitope Database (IEDB): 2018 update. Nucleic Acids Res. 2019; 47:D339–D343

6. Tickotsky N, Sagiv T, Prilusky J, et al. McPAS-TCR: a manually curated catalogue of pathology-associated T cell receptor sequences. Bioinformatics 2017; 33:2924–2929

7. Sidhom J-W, Larman HB, Pardoll DM, et al. DeepTCR is a deep learning framework for revealing sequence concepts within T-cell repertoires. Nat. Commun. 2021; 12:1605

8. Gielis S, Moris P, Bittremieux W, et al. Detection of Enriched T Cell Epitope Specificity in Full T Cell Receptor Sequence Repertoires. Front. Immunol. 2019; 10:

9. Springer I, Tickotsky N, Louzoun Y. Contribution of T Cell Receptor Alpha and Beta CDR3, MHC Typing, V and J Genes to Peptide Binding Prediction. Front. Immunol. 2021; 12:664514

10. Peng X, Lei Y, Feng P, et al. Characterizing the interaction conformation between T-cell receptors and epitopes with deep learning. Nat. Mach. Intell. 2023; 5:395–407

11. Montemurro A, Schuster V, Povlsen HR, et al. NetTCR-2.0 enables accurate prediction of TCR-peptide binding by using paired TCRα and β sequence data. Commun. Biol. 2021; 4:1–13

12. Pham M-DN, Nguyen T-N, Tran LS, et al. epiTCR: a highly sensitive predictor for TCR–peptide binding. Bioinformatics 2023; 39:btad284
